# A whole genome screen for HIV restriction factors

**DOI:** 10.1186/1742-4690-8-94

**Published:** 2011-11-14

**Authors:** Li Liu, Nidia MM Oliveira, Kelly M Cheney, Corinna Pade, Hanna Dreja, Ann-Marie H Bergin, Viola Borgdorff, David H Beach, Cleo L Bishop, Matthias T Dittmar, Áine McKnight

**Affiliations:** 1 Centre for Immunology and Infectious Disease, Blizard Institute, Barts and The London School of Medicine and Dentistry, Queen Mary University of London, 4 Newark Street, London E1 2AT, UK; 2 Centre for Cutaneous Research, Blizard Institute, Barts and The London School of Medicine and Dentistry, Queen Mary University of London, 4 Newark Street, London E1 2AT, UK

## Abstract

**Background:**

Upon cellular entry retroviruses must avoid innate restriction factors produced by the host cell. For human immunodeficiency virus (HIV) human restriction factors, APOBEC3 (apolipoprotein-B-mRNA-editing-enzyme), p21 and tetherin are well characterised.

**Results:**

To identify intrinsic resistance factors to HIV-1 replication we screened 19,121 human genes and identified 114 factors with significant inhibition of infection. Those with a known function are involved in a broad spectrum of cellular processes including receptor signalling, vesicle trafficking, transcription, apoptosis, cross-nuclear membrane transport, meiosis, DNA damage repair, ubiquitination and RNA processing. We focused on the PAF1 complex which has been previously implicated in gene transcription, cell cycle control and mRNA surveillance. Knockdown of all members of the PAF1 family of proteins enhanced HIV-1 reverse transcription and integration of provirus. Over-expression of PAF1 in host cells renders them refractory to HIV-1. Simian Immunodeficiency Viruses and HIV-2 are also restricted in PAF1 expressing cells. PAF1 is expressed in primary monocytes, macrophages and T-lymphocytes and we demonstrate strong activity in MonoMac1, a monocyte cell line.

**Conclusions:**

We propose that the PAF1c establishes an anti-viral state to prevent infection by incoming retroviruses. This previously unrecognised mechanism of restriction could have implications for invasion of cells by any pathogen.

## Background

Viruses usurp normal cellular processes to complete their life cycle. Once inside the cell cytoplasm viral RNA is reverse transcribed into single stranded cDNA followed by double stranded (ds)DNA. The dsDNA in cells forms a pre integration complex (PIC) which includes viral proteins and interacts with numerous cell components. Eventually the PIC is transported into the nucleus for host DNA integration.

The use of small-interfering RNA (siRNA) screens has greatly extended our knowledge of the cellular processes hijacked by viruses for infection and the components needed by HIV to facilitate these early steps in replication [1-4]. For example TNPO3, was identified by two screens to be a required for a replication step in the HIV life cycle [1,2]. TNPO3 was later shown to facilitate nuclear import of the PIC [5].

Host cells, however, have evolved intrinsic resistance factors to mitigate viral replication. Several host restriction factors have been identified that prevent the progression of HIV replication during the early phase of the life cycle. The best characterised of these are encoded by the TRIM5α and the APOBEC gene families [6,7]. APOBECs interact with the nascent DNA during reverse transcription [6]. TRIM5α interacts with incoming viral capsids (CA) resulting in premature disassembly [7]. TRIM28/KAP1 has recently been shown to restrict integration of HIV-1 [8]. p21(Waf1/Cip1/Sdi1) (p21) was identified to act during or after reverse transcription [9,10]. SAMHD1 acts prior to integration, possibly by degrading or preventing the accumulation of HIV DNA [11]. Another restriction factor Tetherin (BST-2/CD317) acts post integration to prevent viruses from leaving the cell during the budding stage of the life cycle [12].

To detect intrinsic anti-viral restriction factors acting at the early, post fusion stages of HIV-1 replication, HeLa-CD4 cells were transfected with an siRNA library targeting 19,121 human genes and then challenged with an HIV-1^89.6R ^pseudovirus carrying a GFP reporter gene (HIV-1 *gag/pol/tat *and *rev*, HIV-2 MCR Env). The negative factors identified perform a diverse range of cellular activities. Those with known function are involved in receptor signalling, vesicle trafficking, transcription, apoptosis, cross-nuclear membrane transport, meiosis, DNA damage repair, ubiquitination and RNA processing. Our screen for anti-HIV factors can serve as a platform to understanding the host's adaptation viral infection.

## Results

### System Setup

**Figure 1:**
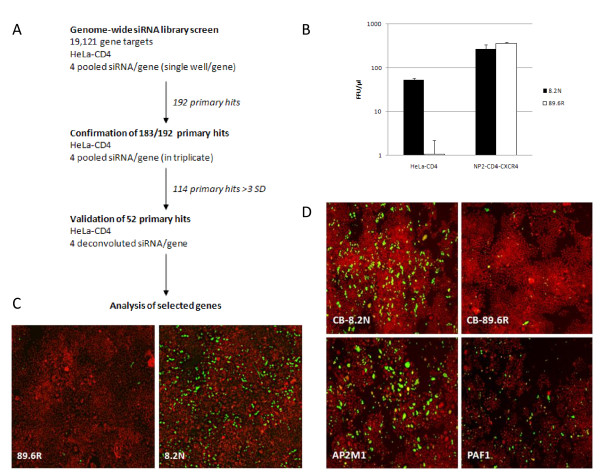
**siRNA screen setup**. **1A **Screen strategy and results. **1B **Infectious units/μl of HIV^8.2N ^and HIV^89.6R ^virus stocks following challenge on HeLa-CD4 and NP2-CD4-CXCR4 cells. Results are mean ± SD of a representative experiment performed in triplicate. **1C **GFP+ foci following virus challenge of HeLa-CD4 cells of HIV^89.6R ^and HIV^8.2N^. Green, virus; red, cells. **1D **siRNA knockdown of AP2M1 and PAF1 rescues infection of HeLa-CD4 cells by HIV^89.6R ^compared with a negative control siRNA (CB). HIV^8.2N ^is the non-restricted positive control. % infection: AP2M1 3.57%, PAF1 0.65%, CB 0.0004%. Green, virus; red, cells.

To detect human cellular restriction factors that operate at the early stages of HIV-1 replication, we developed a single round infectious HIV pseudotype assay to siRNA screen HeLa-CD4 cells. The HIV pseudotype HIV^89.6R^, has an HIV-2 Env MCR (derived from the primary isolate prCBL-23). HeLa-CD4 cells contain ectopically expressed CD4 but naturally express the co-receptor CXCR4. Both receptors are used by HIV^89.6R ^to enter cells. HIV^89.6R ^was evaluated for tropism in HeLa-CD4 cells. Although HIV^89.6R ^replicates efficiently on NP2-CD4-CXCR4 cells it is restricted on HeLa-CD4 cells (Figure 1B) while HIV^8.2N ^grows equally well on both cell types and was used as a positive control for viral replication and to monitor the GFP expression and siRNA effects (Figure 1C). The viral pseudotypes HIV^89.6R ^and HIV^8.2N ^are only capable of a single round of infection so the number of GFP expressing cells is equivalent to virus infectious units (or focus forming units, FFU). An increase in infectious units after siRNA gene knockdown followed by virus challenge after 72 hours indicated rescue of viral replication.

To optimise the screen we used negative control siRNAs targeting cyclophilin B (CB; siGLO), PLK1 and GFP. The reverse transfection protocol was almost 100% efficient, CB protein expression was reduced by 60%, the PLK1 siRNA (cell killer control) reduced cell number by more than 99% and GFP siRNA reduced GFP intensity by 67.8% (data not shown). siRNAs against the CD4 receptor and the nuclear importin TNPO3 were used to test the effects of the siRNAs on the inhibition of non-restricted HIV^8.2N ^infectivity. The results show that the infection was reduced by 95.8% and 93.0%, respectively (Additional file 1, A1).

### Primary siRNA screen

Figure 1A shows a schematic representation of the screen results. Target HeLa-CD4 cells were transfected in 384 well plates with a whole genome siRNA library (19,121 human genes, four pooled siRNAs per gene, 30 nM). siRNA targeting CB served as a negative control (n = 16 per plate). HeLa-CD4 cells transfected with HIV^8.2N ^served as a positive control for the GFP readout. The screen was performed with 30 nM total siRNA concentration to minimise off-target effects. After 72 hours, transfected cells were challenged with HIV^89.6R ^- which carries a GFP reporter gene (HIV-1 *gag/pol/GFP*). Five days post siRNA transfection, cells were stained with DAPI and Cell Mask (Invitrogen) to enable segmentation of GFP+ and GFP- foci. Images were collected on the IN Cell 1000 microscope (GE Healthcare). The images were quantified by IN Cell Developer software (GE Healthcare) to generate the total cell number and GFP+ foci per well (equivalent to FFU).

**Figure 2:**
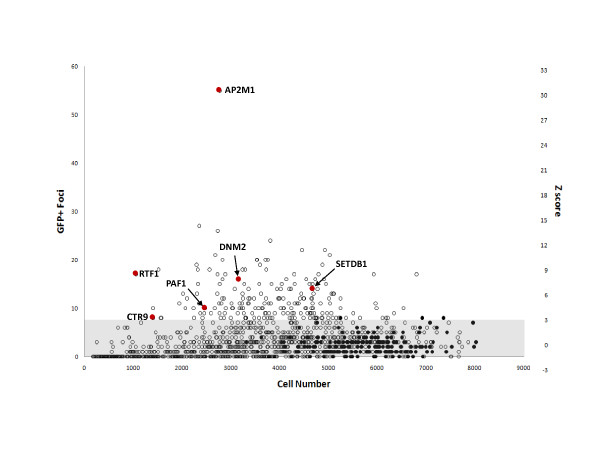
**Validation of screen positive results**. Distribution of rescued HIV^89.6R ^infection determined by GFP+ foci/well (left Y axis) with Z-scores (right Y axis) for the siRNA library (open circles) compared with CB control siRNA (closed circles). AP2M1, DNM2, SETDB1 and genes associated with the PAF1c CTR9, PAF1 and RTF1 are highlighted. GFP+ foci more than 3 SD (equivalent to 7.43 foci) from the average of control infections are shown.

Statistical analysis was performed for each of the 61 plates. Wells containing > 3SD foci of infection compared to the CB plate mean were confirmed by visual inspection. A robust Z-score of > 3 (which equates to a > 3SD difference from the mean; equivalent to rescue of more than 1.2 × 10^3 ^FFU/ml) was deemed significant (Figure 2A). Two examples (AP2M1 and PAF1) of the 'positive hits' are shown in Figure 1D alongside wells challenged with the restricted HIV^89.6R ^and the unrestricted HIV^8.2N^. The results in Figure 2 show that 192 genes increased the GFP foci by more than 3SD from the controls (Additional file 2 contains a complete list of genes and their corresponding Z-scores, A2).

### Secondary screen

Next, we performed a secondary validation screen for 183 of the 192 candidates from the primary screen. Transfection of the original pooled siRNAs was performed in triplicate and the same Z-score threshold applied. This yielded 114 genes, equivalent to a 62.3% validation rate (Additional file 3). Ingenuity Pathway Analysis (IPA, http://www.ingenuity.com) was used to analyse the 114 confirmed genes. Network, functional and pathway analysis was carried out with the IPA software. The identified genes are involved in a wide variety of networks, such as cell signalling, molecular transport, nucleic acid metabolism, cell cycle, DNA replication, and recombination and repair. Additional file 4 summarises the analysis (see also additional file 5, Table including gene ontology (GO) terms, A5). Functional analysis shows the participation of skeletal development, cell signalling, molecular transport, nucleic acid metabolism, cell cycle, cell-to-cell signalling and interaction, lipid metabolism, renal and urological disease, reproductive system development, cell-mediated immune response, DNA replication, RNA post-transcriptional modification, recombination and repair, antigen presentation and the humoral immune response. Pathway analysis reveals possible pathways in which these genes may be involved and include the CXCR4 signalling pathway, virus entry via the endocytic pathway, clathrin-mediated endocytosis and dendritic cell maturation.

### Validation by individual siRNAs

We chose 52 genes (either with high Z-scores or because their functions were related) to validate with multiple individual siRNAs. The four siRNAs from the original screening pool were individually tested at 30 nM using the same protocol as the secondary screen. If cell toxicity was observed, siRNA concentrations were adjusted. This analysis revealed that 100% (52/52) of the genes were confirmed with at least one siRNA, while 53.8% (28/52) re-scored with two or more siRNAs (Table 1).

**Table 1:** Number of individual siRNA able to rescue HIV-1 infection in HeLa-CD4 cells.

4/4	3/4	2/4	1/4
AP2M1	COX18	ALDH8A1	BCAR1
C3orf63	DNM2	ALX3	C11orf38
CTR9	RPRD2	BCYRN1	C15orf27
PAF1	SPCS2	C11orf38	DSP
RTF1		CCDC53	EVI2B
SETDB1		ELF3	FUT1
		IL1F9	GANC
		LOC388955	GCNT3
		MARCH8	ICAM4
		MKRN3	INO80B
		OPTC	KCNG1
		PELP1	KCNN1
		POLB	KLK3
		R3HDML	LRRC24
		RAPGEF3	POP5
		SHE	RMI1
		SLC9A3R2	SEBOX
		SPAG16	SLC35B1
			SNORD114-31
			SNORD115-32
			TMEM209
			TRIM27
			WSB2
			ZNF761

Four genes; COX18, DNM2, RPRD2 and SPSC2, were re-scored with 3/4 siRNAs. There were six genes for which transfection with all four siRNAs enhanced virus infection. We focussed on these genes in further functional analysis. Three of these, CTR9, PAF1 and RTF1, all belong to the human PAF1 complex [13].

### AP2M1 and dynamin (DNM2) restrict HIV-2 but not HIV-1

Down modulation of AP2M1 and DNM2, both involved in endocytosis, increased infectivity of HIV^89.6R ^by 3.1 × 10^4 ^and 1.6 × 10^4 ^infectious unit/ml respectively (Z score 30.5 and 7.9; Figure 2 and 2A3) and were chosen for further validation and analysis.

**Figure 3:**
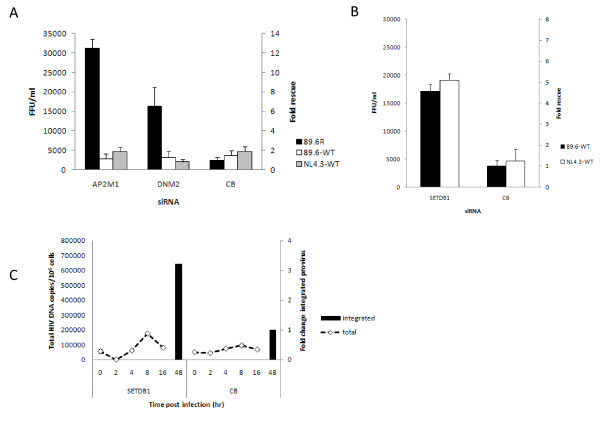
**Analysis of selected genes**. **3A **AP2M1 and DNM2 siRNA knockdown rescues infection of HeLa-CD4 cells by pseudotyped HIV^89.6R ^and wild type HIV-1^89.6wt ^and HIV-1^NL4.3wt^. The y-axis denotes both the number of infectious viruses rescued (FFU/ml; left) and fold rescue compared with the CB control (right). FFU/ml results are mean ± SD. **3B **SETBD1 siRNA knockdown rescues infection of HeLa-CD4 cells by replication competent HIV-1^89.6wt ^and HIV-1^NL4.3wt ^(MOI 0.7). FFU/ml results are mean ± SD. **3C **Knockdown of SETDB1 results in enhanced HIV-1 RT products - total (0-16 hr pi) and integrated proviral DNA (*Alu*-*gag*, fold change, 48 hr pi). Control wells containing inhibitors were negative for HIV-1 DNA. HIV-1 DNA copies are normalised to genomic GAPDH and presented per million cells. Results are mean ± SD of a representative experiment performed in duplicate.

We treated HeLa-CD4 cells with siRNA to AP2M1 and DNM2 and challenged with either the pseudotype HIV^89.6R ^or two wild type HIV-1 strains, T-cell tropic HIV-1^NL4.3wt ^and the dual tropic HIV-1^89.6wt ^which can infect both T-cells and macrophages. Interestingly, Figure 3A shows that even though AP2M1 and DNM2 knockdown rescued HIV^89.6R ^by 12.6 and 6.5 fold, respectively there was no rescue of wild type HIV-1. Indeed none of the four siRNAs to AP2M1 or dynamin (DNM2) rescued HIV^89.6wt ^or HIV^NL4.3wt ^viruses (data not shown). The major difference between the HIV pseudovirus and the wild type HIV-1 viruses is that HIV^89.6R ^is pseudotyped with an HIV-2 Env. The Env of the HIV-2^MCR ^virus is a determinant of a post-entry restriction, Lv2 [14-17]. Although HIV-1 viruses are generally unrestricted when entering cells via an endocytic dynamin dependent route [18] we have shown that if cellular endocytosis is blocked HIV-2^MCR ^virus is rescued from Lv2 restriction [14,15,17]. More specifically we have recently shown that down modulation of AP2M1 by siRNA or dominant negative inhibition of DNM2 can rescue HIV-2^MCR ^(envelope mediated) inhibition by Lv2 [16,19]. Thus our results here showing rescue of the HIV-2 but not HIV-1 envelope mediated entry is consistent with these previous observations. The identification of AP2M1 and DNM2 in our primary screen further demonstrates the stringency of the primary screen.

### SETDB1 inhibits HIV-1 replication at a step prior to integration

Surprisingly, all siRNA targets to SETDB1 resulted in rescue of HIV^89.6R ^replication. SETDB1 is a histone methyl transferase and along with heterochromatin protein 1 (HP1) and NuRD, a histone deacetylase (HDAC), is recruited by TRIM28 (also known as KAP1, KRAB-associated protein 1) to inhibit replication of endogenous retroelements during embryonic development [20]. TRIM28 has also been reported to restrict replication of the gamma retrovirus M-MLV in cells by inhibition of proviral gene expression [21]. Our result was a surprise because there is no previous evidence suggesting that HIV-1 is targeted prior to integration. Indeed our screen used an HIV pseudovirus capable of only a single round of infection so genes enhancing post integration events are unlikely to be detected. To investigate we selected SETBD1 for further analysis.

SETDB1 had a Z-score of 6.8 in the initial screen. Down modulation of SETDB1 enhanced the infection of both HIV^89.6R ^and wild type HIV-1 (8.8 × 10^3 ^and 1.7-1.9 × 10^4 ^FFU/ml) (A3 and Figure 3B). To probe the time point in the viral replication that was impeded by the expression of SETDB1 proteins we PCR-amplified total viral DNA (*gag*) to detect progression and completion of reverse transcription after treatment with SETDB1 siRNA. An *Alu*-PCR was established to detect integrated proviral DNA. Analysis of the effect of SETDB1 knockdown on the levels of total HIV-1 DNA showed no apparent difference while there was a 3.2 fold increase in integrated proviruses (Figure 3C).

Thus our results strongly suggested that SETDB1 is also involved in inhibition of HIV-1 replication. Consistent with this hypothesis Allouch *et al. *report that TRIM28 indeed restricts replication of HIV-1 integration through recruitment of HDAC [8]. The molecular details will be worth pursuing.

### PAF1 complex expression renders cells in an antiviral state

A striking observation is that enhancement of infection results after independent knockdown of 3 components of the PAF1 complex (PAF1c) [22-24], PAF1, CTR9 and RTF1 (A3, Z scores from the initial screen of 4.4, 3.3 and 8.5 respectively). These were chosen for further analysis and 2 additional components of the PAF1 complex, CDC73 and LEO1, were included in subsequent investigations.

To further confirm the specificity of the siRNAs we tested each of the PAF1c siRNAs for their effect on expression of each other. We find that the siRNA to PAF1, CTR9 and RTF1 reduced their own mRNA levels and not each other's (data not shown).

**Figure 4:**
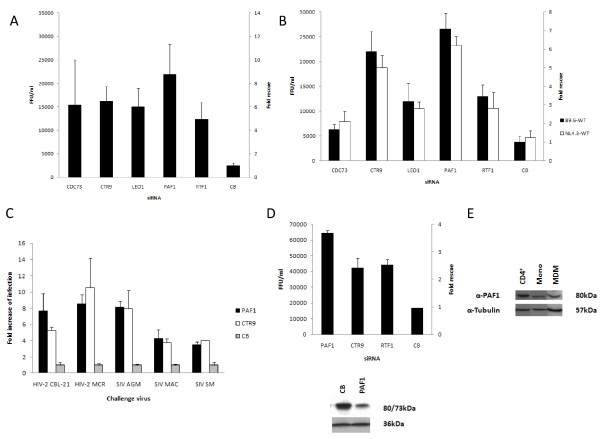
**Anti-viral activity of the PAF1 complex**. **4A **CDC73, CTR9, LEO1, PAF1 and RTF1 siRNA knockdown rescues infection of HeLa-CD4 cells by HIV^89.6R ^pseudotype. CDC73 and LEO1 were additionally deconvoluted and 4/4 and 3/4 siRNA respectively were able to rescue HIV-1^89.6R ^infection in HeLa-CD4 cells. The y-axis denotes both the number of infectious viruses rescued (FFU/ml; left) and fold rescue compared with the CB control (right). FFU/ml results are mean ± SD. **4B **PAF1c siRNA knockdown rescues infection of HeLa-CD4 cells by replication competent HIV-1^89.6wt ^and HIV-1^NL4.3wt ^(MOI 0.7). FFU/ml results are mean ± SD. **4C **Knockdown of PAF1 and CTR9 rescues HIV-2^CBL-21^, HIV-2^MCR^, SIV^AGM ^(African Green Monkey; 3084), SIV^MAC ^(Macaque; 32H) and SIV^SM ^(Sooty Mangabey; B670) infection. Results are fold increase compared with CB and are mean ± SD of at least 3 representative experiments. **4D **Knockdown of PAF1, CTR9 and RTF1 in MM1 cells results in rescue of HIV-1 infection of replication competent HIV-1^89.6wt^. Results are mean ± SD of a representative experiment. Transfection of MM1 cells with PAF1 siRNA results in partial knockdown. **4E **Western blot analysis of primary cell lysates (CD4^+ ^T cells, monocytes and macrophages (MDM)) showing protein levels of PAF1.

Figure 4A shows that in addition to PAF1, CTR9 and RTF1 identified in the screen, knockdown of the remaining 2 components of the PAF1c, LEO1 and CDC73 also rescued infection of HIV^89.6R^. LEO1 and CDC73 were false negatives in the primary screen. Next we tested the effect of inhibition of expression of these factors against the wild type viruses HIV-1^NL4.3wt ^and HIV-1^89.6wt^. Figure 4B shows that knockdown of any one of all five components of the PAF1c rescued infection of wild type viruses by 1.7-7.2 fold (equivalent to 6.2 × 10^3^-2.7 × 10^4 ^FFU/ml).

To determine whether the anti-viral effect observed was restricted to HIV-1 we tested for activity against HIV-2 and SIV isolates. We used siRNA to two PAF1c components, PAF1 and CTR9. Figure 4C shows that inhibiting expression of either of these proteins resulted in rescue of HIV-2 (CBL-21 and MCR) and SIV (African Green Monkey, Macaque and Sooty Mangabey) by 4-10 fold.

We wanted to test if there was an effect of PAF1c in cells of more physiological relevance to HIV-1 infection. Figure 4D shows that treatment of the differentiated monocytoid cell line MonoMac1 (MM1) with siRNA targeting PAF1, CTR9 and RTF1 resulted in rescue of HIV-1^89.6wt ^(2.5-4.7 × 10^4 ^FFU/ml, 2.5-4 fold). In Figure 4E Western blot analysis reveals that PAF1 is expressed in primary CD4+ T-cells, monocytes and monocyte derived macrophages. Attempts however to knock down expression in these cell types resulted in cytotoxicity.

**Figure 5:**
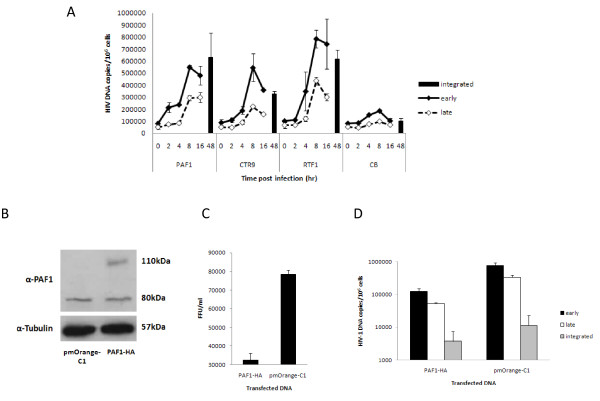
**5A Knockdown of PAF1, CTR9 and RTF1 results in enhanced HIV-1 RT products - early and late (0-16 hr pi) and proviral DNA (*Alu*-*gag*, 48 hr pi)**. Control wells containing inhibitors were negative for HIV-1 DNA. HIV-1 DNA copies are normalised to genomic GAPDH and presented per million cells. Results are mean ± SD of a representative experiment performed in duplicate. **5B **Transfection of a PAF1-HA expression construct into HeLa-CD4 cells results in increased levels of PAF1. The PAF1-HA clone possesses an HA and an orange tag (~30 kDa, accounting for the 110 kDa band detected). **5C **Over-expression of PAF1-HA resulted in a decrease in FFU/ml following challenge with HIV-1^89.6wt^. Results are representative of multiple experiments and are mean ± SD. **5D **Over-expression of PAF1-HA results in decreased RT products - early and late (8 hr pi) and proviral DNA (integrated, 48 hr pi). HIV-1 DNA copies are normalised to genomic GAPDH and presented per million cells. Results are mean ± SD of a representative experiment.

There are a number of recognised steps in the early phase of retroviral replication that restriction factors might target. To determine the time point that viral replication was blocked due to the action of PAF1c proteins we PCR-amplified viral strong-stop DNA (ssDNA) to detect the initiation of reverse transcription and full length viral cDNA to detect progression and completion of reverse transcription after treatment with various siRNAs. *Alu*-PCR was used to detect integrated proviral DNA. Figure 5A shows that knockdown of PAF1, CTR9, and RTF1 resulted in enhanced viral ssDNA levels as well as full length cDNA of 3-7 fold more than CB treated cells indicating that a block to reverse transcription was relieved. An alternative explanation is that RT products may be less susceptible to degradation. There was also enhanced proviral integration (*Alu*-PCR) of full length cDNA transcripts (Figure 5A). Thus our data support the conclusion that the expression of the PAF1 complex induces an anti-viral state which results in blocking viral replication during the early events from post entry to integration of proviral DNA. To further test this hypothesis we made an HA-tagged expression construct of PAF1, transfected it into HeLa-CD4 cells and challenged with HIV-1^89.6wt ^after 24 hours. Figure 5B shows Western blot analysis of transfected cells expressing the expected HA-tagged PAF1 (PAF1-HA) construct at 110 kDa. The level of endogenous PAF1 (80 kDa) is similar to the vector control (pmOrange-C1). Figure 5C shows that even though the transfection efficiency was moderate (30% by visualisation of a simultaneously transfected GFP plasmid; data not shown) inhibition of HIV-1^89.6wt ^was observed. We further analysed the levels of inhibition of various stages of reverse transcription and integration of proviral DNA. Figure 5D shows a reduction of all viral transcripts and provirus. Early and late RT products are decreased by 6.3 and 2.8 × 10^5 ^copies/10^6 ^cells, respectively, while there are 7.7 × 10^3 ^fewer integrated proviruses/10^6 ^cells in samples that contain higher amounts of PAF1.

Thus our data so far support a role for the PAF1c in the innate defence of host cells against viral infection. We sought to gain some insight into the mechanism of PAF1c's antiviral action. PAF1c activity has been implicated in a number of cellular processes including gene expression and transcription, mRNA elongation and stability and cell cycle control [22].

**Figure 6:**
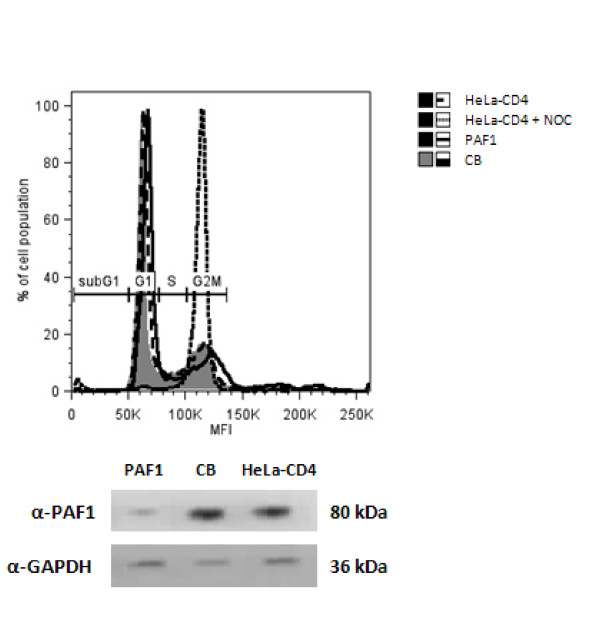
**Silencing of PAF1 does not affect cell cycle progression**. HeLa-CD4 cells transfected with PAF1 or CB siRNA were compared to nocodazole (NOC) treated controls. Cell cycle profiles were determined by flow cytometric analysis of PI-labelled cultures. Western blot confirms PAF1 silencing compared to CB control.

We tested whether the cell-cycle arrest activity of PAF1 could account for the rescue of infectivity observed. PAF1 knockdown can result in early entry into S phase and later transition out of G2 phase [25]. Knockdown of PAF1 therefore would have the same outcome as the expression of the viral gene *vpr *which induces cell-cycle arrest at G2/M phase [26] and facilitates viral replication. Figure 6 demonstrates however that in the target cells used in this study down modulation of PAF1 does not affect cell cycling.

PAF1 is also suggested to be involved in mRNA stability, surveillance and degradation. It directly interacts with SKI8 (WDR61) which forms a complex with SKI2 and SKI3 [24]. WDR61 was included in subsequent follow up experiments and weak rescue of infection was observed (2 fold, not shown). In yeast the SKI complex is required for 3'-5' mRNA decay in exosomes which are regions in the nucleus and cytoplasm that specialise in RNA degradation [22,27]. Finally, PAF1 is known to be involved in transcription of DNA and elongation of mRNA through its association with RNA polymerase II during these processes [28]. It is also required for mono-ubiquitination and methlylation of histones which affect gene expression [29]. Indeed it has been shown recently [30] that PAF1 along with P-TEFb, AF9, AFF1, AFF4, ELL and ENL is recruited to the HIV LTR by Tat for optimal promoter activation. The pseudovirus used in our screen is only capable of single round of infection so it is unlikely that post-integration events are detected. Regardless we confirmed that PAF1 was not suppressing viral gene expression. qPCR analysis of viral genes after treatment of cells with siRNA to PAF1, CTR9 and RTF1 did not result in enhanced expression of viral gene transcripts (data not shown). Thus we hypothesised that PAF1c's action was through the expression of anti-viral factors. We tested whether PAF1c induces an anti-viral state through activation of transcription of known anti-HIV factors. Following treatment of HeLa-CD4 cells with PAF1 siRNA, the mRNA levels of p21, APOBEC3G, Tetherin, TRIM5α, SAMHD1 and TREX1 were determined by qPCR. No difference was seen in the expression of these restriction factors when compared with untreated cells (data not shown) suggesting that PAF1 does not exert its anti-viral activity through the action of these known restrictive genes.

We conclude that PAF1c induces an anti-viral state in cells and inhibits infection of HIV-1, 2 and SIV by blocking progression of the early events of infection during reverse transcription and up to integration. Our data suggest that the anti-viral effect of PAF1 is not mediated through control of the cell cycle, enhancement of expression of viral transcripts, enhancement of viral genome stability or enhancement of transcription of known anti-viral restriction factors such as p21.

In summary we report here the results of an siRNA screen to identify restriction factors to HIV-1 replication by knockdown of expression of 19,121 human genes. We identified 114 genes that affect a wide range of cellular activities. Here we specifically characterised the involvement of AP2M1, DNM2, SETDB1, PAF1, CTR9 and RTF1 in defence against retroviral invasion.

## Discussion

A fairly comprehensive picture of the positive interactions between HIV-1 and host cell proteins has been established in recent years using siRNA screens that reveal how HIV-1 exploits the host cellular machinery [1-4]. Here, we report 114 factors which, in contrast to positive factors, are involved in intrinsic immunity to establish an anti-viral attack in human cells. In this paper we refer to intrinsic immune restriction factors as cellular factors that are constitutively expressed or can be generated in cells in the absence of help from other arms of the immune system. Such factors however are unlikely to act in isolation of the immune system and may indeed be the initial trigger for innate and adaptive immune responses in the host. The proteins identified with known functions are associated with a vast array of cellular processes such as receptor signalling, vesicle trafficking, transcription, mRNA processing, DNA/RNA surveillance, cross-nuclear membrane transport and ubiquitination.

In our screen individual silencing of 114 genes rescued infectivity of HIV-1 by more than 10^3 ^FFU/ml and for some genes as many as 2 × 10^5 ^infectious units/ml (1.6-12.6 fold). The 52 most potent siRNAs 'hits' were further validated using 4 unpooled siRNAs. Rescue of viral infection by 2 or more of the 4 siRNAs was observed for 28 genes. Of these, AP2M1, DNM2, CTR9, PAF1, RTF1 and SETDB1 were further characterised for inhibition of HIV infection.

At the time of starting this screen only APOBEC3G/F and rhesus macaque TRIM5α proteins were known as factors that restrict HIV-1 replication. APOBECs are packaged into virus particles in the producer cells but act on the next round of infection in the new target cell [6]. The HEK 293T cells used to produce the pseudovirus in our screen do not express APOBECs [6] so identification of these proteins was not expected. Neither did we identify TRIM5α because it is a species specific restriction factor and the human protein does not significantly affect HIV-1 [7,31]. Since the screen was completed two other factors have been reported; p21 acts during reverse transcription and is active in stem cells, macrophages and CD4+ lymphocytes [9,10,32] while SAMHD1 expression is confined to dendritic and myeloid lineage cells [11]. Tetherin, which acts at the late phase of the viral life cycle prevents viral budding [12], was not identified in this screen because we probed the siRNA library with an HIV pseudotype that is infectious for only a single round to the integration of provirus and GFP expression.

Using a simple model of viral restriction, intrinsic restriction factors can be loosely divided into; detectors of invasion, messengers, mediators and effectors that either prevent infection of the virus or, in the case of interferon (IFN) signalling, alert neighbouring cells. IFN activation of the JAK-STAT pathway ultimately results in the expression of anti-viral genes. For example the APOBEC and TRIM gene families are induced by type I IFNs [33].

After entry into the cell cytoplasm HIV-1 uncoating involves the disassembly of the viral matrix (MA) and capsid (CA) and release of the genomic RNA and the associated proteins. Thus these viral components could potentially be seen by the host cell as 'non-self' molecules. Pattern Recognition Receptors (PRRs) or viral sensors recognise distinct pathogen associated molecular patterns (PAMPs). For example, recently TRIM5α was shown to be a PRR that recognises the viral CA eventually resulting in immune signalling and AP-1 and NF-κB activation [34]. Of similar interest, silencing of COX18 (a cytochrome c oxidase assembly protein) resulted in rescue (3.3 fold). COX18 is essential for the production of COX2 (PTGS2) [35] whose expression is attenuated by TRIM5α knockdown [34]. It seems unlikely that COX18 itself is a direct PRR. It is more plausible that COX18 is an essential downstream component that through its interaction with COX2 may commission or interact with one or more 'effectors' of restriction.

During and after CA disassembly the viral nucleic acids and those produced during reverse transcription, RNA fragments, DNA and DNA:RNA hybrids may become exposed to cytoplasmic PRRs. Proteins that interact with nucleic acids might also play a role in pattern recognition. Our screen did not identify any of the classical RNA-recognising PRRs such as toll like receptors TLR3, 7 or 8 or RIG-1 like receptors RIG-1 or MDA-5 [36]. The best characterised DNA receptor DAI (DNA dependent activator of IFN regulatory factors) responds to adenoviral DNA [37]. In this screen silencing of POLB polymerase (DNA directed) rescued viral infection (3.5 fold). POLB rescue was validated with 2 individual siRNAs. POLB is involved in DNA damage repair and performs base excision repair required for DNA maintenance, replication, recombination [38]. Interestingly, POLB is specifically up regulated after infection of cells with human herpes virus 16.

Once PAMPS are engaged multiple signalling cascades are activated and establish an anti-viral state [39]. One potent hit IL1F9, an interleukin cytokine, is switched on by IFNγ. Interestingly expression of this gene is also induced after herpes simplex virus infection-1 [40]. Signal induced antiviral responses will also use *de novo *transcription and translation to establish an anti-viral environment. Three transcription factors ALX3, ELF3 and PELP1 (3, 4 and 3.4 fold, validated 2/4 siRNAs) identified in the screen could be involved in this process.

The screen identified a number of genes that are involved in ubiquitination of proteins. Conjugation of the ubiquitin monomer to proteins is mediated by three families of enzymes; E1, E2 or E3 substrate specific ubiquitin ligases [41]. E3 enzymes are critical and interact with both E2 and substrate. Generally, ubiquitination leads to the degradation of proteins by the proteasome. In viral restriction ubiquitination could be involved in the degradation process of either viral proteins or recycling of restriction proteins. The process of ubiquitination is involved in TRIM5α mediated restriction [7]. TRIM5α has E3 ligase activity. When E2 binds to the RING domain E3 ligase activity of TRIM5α transfers the ligase from E2 to TRIM5α [42]. HIV-1 Vif appears to use ubiquitination as a means of preventing the action of APOBEC proteins, by targeting the Cul5-elongin B elongin C-Rbx ubiquitin ligase. This results in the polyubiquitination of APOBEC3G which is then degraded by proteosomes [43-47]. A strong 'hit' in our screen was the E3 ubiguitin ligase MARCH8 (RNF178, MIR) [48] whose knockdown resulted in rescue (4.6 fold and 2/4 unpooled siRNA). A second strong hit was MKRN3 (markorin 3, ring finger protein, 3) where 2 individual siRNAs rescued infection (4.7 fold). Silencing of RNF19A, TRIM25 and TRIM27, which all belong (or are related) to E3 ligases also rescued infectivity (3-3.5 fold between 1 × 10^3 ^and 3 × 10^3 ^FFU/ml). Interestingly TRIM25 is essential for RIG-1 mediated IFNβ production and antiviral activity [49]. However of the latter three genes only TRIM27 was validated with individual siRNA with only 1 out of 4 siRNAs confirming the restriction phenotype. Additionally knockdown of WSB2, a bridge-protein which connects substrate-binding domains and E3 ubiquitin protein ligases had a modest rescue (3.2 fold, 1 of 4 individual siRNA).

### Nuclear import

Once production of the viral cDNA is complete it must be transported to and enter the nucleus through specialised nuclear pores using host cell transport mechanisms [50]. CA appears to remain associated with the PIC for some time following its production [51,52] but disassociates from the reverse transcription complex prior to nuclear entry [53]. Nuclear trafficking is likely to be another "check point" for the host cell. A truncated protein CPSF6 (cleavage and polyadenylation factor) a member of the S/R family protein impairs nuclear entry of PIC [54]. This restriction can be overcome by CA mutants. In our study invalidation of NPIP (nuclear pore complex interacting protein) resulted in enhanced infection. The function of NPIP is not clear, though it co localises with NUP62, a protein needed for HIV PIC transport from cytoplasm to nucleus [2].

### RNA species

We might expect that some retroviral elements would be involved in anti-viral defence. Friend-virus-susceptibility factor (Fv-1) a gag region of an endogenous retrovirus [55], restricts murine leukaemia virus (MLV) at a stage after cellular entry and before integration [56]. MLV CA is the target for Fv-1 [57-59]. We did not identify endogenous retroviral genes in this screen. However BCYRN1 which is believed to be retropositionally generated [60] was identified (5.8 fold, validated 2/4). BCYRN1 belongs to a family of interspersed repetitive DNA sequences and encodes a neural small untranslated non-messenger RNA. The specific neuronal expression of BCYRN1 is intriguing. Immune privileged sites such as the brain may rely more heavily on innate immune factors to prevent viral infection. The tissue specificity of BCYRN1 implies that it is unlikely to play a role in HIV-1 pathogenesis.

### Genes with no obvious role

A number of genes that currently have no tentative functional role in retroviral defence were identified in the primary screen and validated with at least 2 individual siRNAs: SPSC2 (removes signal peptides from proteins as they translocate into the endoplasmic reticulum lumen), R3HDML (a serine protease inhibitor), SPAG16 (a component of microtubules) and RAPGEF3 (Rap guanine nucleotide exchange factor 3). OPTC is a protein that is associated with the extracellular matrix in the eye. ALDH8A1 is an aldehyde dehydrogenase and LOC388955 (PREL1 domain-containing protein 1) is a mitochondrial pseudogene. A functional role cannot be precluded as genes are frequently found to play a practical role in cellular processes far removed from those with which they were originally associated. Several genes, C3orf63, RPRD2, C11orf38, SLC9A3R2 and SHE had no identified function. Further studies on these genes may shed light on currently poorly understood biological processes.

### Endocytosis genes AP2M1 and DNM2

Some factors identified in our screen might have been predicted: AP2M1 and DNM2, two components of the endocytic pathway were identified in the primary screen to restrict infection of cells by the pseudovirus but not by wild type HIV-1 viruses. This observation can be explained - we previously showed HIV-1 and 2 viruses can be blocked post-entry by a restriction factor Lv2 but only if the virus was delivered to the cytoplasm using a specific endocytic route mediated by an HIV-2 virus MCR envelope [14,16,17]. The entry route is specific to the HIV-2 envelope because pseudoviruses with VSV-G are rescued [15]. We recently showed that blocking entry of HIV-2 MCR virus by an AP2M1/DNM2 dependent route rescued Lv2 restriction. Unlike the wild type HIV-1 viruses, the HIV-1 pseudovirus used in our screen was generated with an Env derived from HIV-2 MCR. So inhibition of the pseudovirus (but not the wild type HIV-1) would be expected to be rescued by siRNAs to AP2M1 and DNM2.

Other factors involved in endocytosis will be interesting to probe with respect to the Lv2 restriction. CCDC53 (coiled-coil domain containing protein), a component of the WASH complex, is present on the surface of endosomes and recruits the Arp2/3 complex for actin polymerisation [61]. Two GTPases (or associated proteins) were identified: RAPGEF3 (Rap guanine nucleotide exchange factor 3) and RAB37 (small GTPase that regulates vesicle trafficking) and knockdown of either resulted in a 3.7 or 2.8 fold rescue. It will be interesting to further evaluate their role in Lv2 restriction. However, apart from vesicle trafficking, GTPases regulate a myriad of cellular functions including signal transduction and cytoskeletal organisation.

### SETDB1

We did not predict that silencing of SETDB1 (SET domain, bifurcated 1) would rescue replication of HIV. SETDB1 is involved in TRIM family protein TRIM28 (also known as KAP1) restriction of both exogenous MLV and endogenous retrovirus expression post integration in murine cells [21]. SETDB1, a histone methlytransferase, the NuRD histone deacetlyase complex (HDAC) and heterochromatin associated protein HP-1 are targeted to proviral DNA by TRIM28 to repress transcription. Furthermore, when transfected into human cells, SETDB1 increases HIV-LTR transactivation in conditions where Tat levels were suboptimal [62] but no function in the viral life cycle prior to integration had been previously described. Our screen with single round pseudotype virus should only identify genes involved in the early phase in HIV replication and not those post integration. Surprisingly, SETDB1 silencing rescued wild type HIV-1 replication by almost 2 × 10^4 ^FFU/ml (4.6 fold). Indeed all 4 siRNAs individually silencing SETDB1 resulted in viral rescue and was a gene among our most significant 'hits'. Upon further investigation we found that there was a small increase in reverse transcripts and that there was a relatively strong increase in integrated proviral DNA. Recently, Allouch *et al. *described a similar phenotype for restriction by TRIM28 through recruitment of HDAC [8]. It will be interesting to probe the molecular details of this interaction in greater depth.

### PAF1 complex expression renders cells restrictive to lentiviral infection

Our validation of the PAF1c showed that all of its known components are important for the observed restriction. PAF1c expression restricts HIV-1, 2 and SIV. It restricts infection of a monocytic cell line and is expressed in primary CD4+ T cells, monocytes and macrophages. The restriction results in fewer viral transcripts both in the early and late stage of infection. It also results in less integrated proviral DNA. We are not sure however if this phenotype is due to PAF1's direct action on replicating virus or if its action is through activation of expression of one or more unknown restriction factors.

A role for PAF1 in the cell cycle has been recently demonstrated [25] and studies using an *in vitro *disassembly model have suggested that uncoating requires cell cycle dependent host cell factors [63]. However the cell cycle was unperturbed by inhibition of PAF1 expression in the Hela-CD4 cells we used in the screen suggesting that PAF1's cell cycle activity is not involved in this restriction.

There are two possibilities for the action of PAF1c in viral restriction: First, PAF1 interacts with RNA polymerase II and is proposed to be involved in transcription, elongation and stability of mRNA, surveillance and degradation [24]. Indeed a large quantity of PAF1c is located in the nucleus so it is also possible that its activity in gene transcription may enhance expression of anti-viral factors, some of which are already 'hits' in our primary screen. Additionally we have excluded a role for APOBEC3G, TRIM5α, p21, tetherin and the recently described SAMHD1.

Second, it has also been demonstrated that SKI8 (WDR61) interacts with the PAF1c [24]. SKI8 is also part of the SKI complex which is involved in mRNA decay [24,27]. Given the ability of PAF1/SKI8 to interact with DNA and RNA and the localisation of both these components in the cytoplasm it is possible that PAF1c/SKI8 could act as a PRR. DNA:RNA hybrids or degrading RNA genomes are possible targets. To distinguish between the two possibilities of PAF1's action it may be of use to determine which genes are enhanced in the presence or absence of PAF1 using gene array analysis.

## Conclusions

In summary we describe the identification of 114 genes that are involved in restriction of HIV replication during the early stages of the viral life cycle. Preliminary characterisation of 6 genes AP2M1, DNM2, CTR9, PAF1, RTF1 and SETDB1 confirms their biological role in retroviral restriction. This preliminary characterisation attests to the robustness of the primary screen. It is improbable that the genes identified evolved in resistance to HIV-1 infection. It is more plausible they are active against other viruses or pathogens that invade host cells and will be a platform for a general understanding of innate immune or intrinsic defence against invasion of known and emerging pathogens. Variation in the expression patterns and polymorphisms in these genes may lead to an understanding of the reasons why some individuals are more or less susceptible to specific infectious diseases.

## Methods

### Cells

Culture of HEK 293T, HeLa-CD4, NP2-CD4-CXCR4, MonoMac 1 (MM1) and C8166, and their optimal culture conditions, have been described previously [17,64,65]. Peripheral blood mononuclear cells (PBMC) were prepared from seronegative donors by density-gradient centrifugation (Lymphoprep, Axis-Shield). CD4^+ ^T lymphocytes were isolated using CD4 Microbeads according to manufacturer's instructions (Miltenyi Biotech). Monocyte-derived macrophages (MDM) were prepared by adherence as described previously [66], except that cells were harvested (for monocyte preparations) and replated at 2 × 10^6 ^cells/ml following the initial overnight incubation, and left to differentiate for 7-14 days in RPMI 1640 supplemented with 20% autologous human serum and 20 ng/ml macrophage colony stimulating factor (M-CSF; R&D Systems).

### Plasmids and virus production

The expression plasmid for PAF1 was generated by PCR amplifying the open reading frame from HeLa-CD4 cDNA which was subcloned into the pmOrange-C1 vector (Clontech). Primers are available upon request. Infectious molecular clones for wild type (wt) HIV-1^89.6wt ^and HIV-1^NL4.3wt ^were obtained from the Centre for AIDS Research (NIBSC, UK). Restricted pseudotyped virions (HIV^89.6R^) were generated by combining the transfer vector pCSGW with the restrictive HIV-2 envelope MCR [16,17] and the core construct p8.91-89.6*gag*. For the non-restricted viral particles (HIV^8.2N^), we used MCN Env and p8.2-89.6 core, where the *gag *fragment from p8.91-89.6*gag *was transferred into pCMVΔ8.2 according to the method described [67]. Virus stocks were prepared from infectious full-length and chimeric HIV clones by polyethylenimine (Polysciences) transfection of HEK 293T cells. SIV and HIV-2^CBL-21 ^stocks were grown in C8166 cells.

### siRNA screen

The screen was performed using the siRNA library (QIAGEN, the Human Whole Genome siRNA Set V4.0) which enables gene silencing studies of 19,121 genes from the RefSeq database (NCBI handbook). HeLa-CD4 plated at 600 cells/well in a 384-well plate were transfected with 30 nM siRNA from the library or control siRNA PPIB (cyclophilin B, referred to as CB) using HiPerfect (QIAGEN) and the CyBio Vario (CyBio, Germany) liquid handling system. The system was optimised using siRNAs to CB and GFP. The siRNAs against receptor CD4 and the nuclear importin TNPO3 reduced infectivity of HIV^8.2N ^by 95.8% and 93.0%, respectively.

Plates were incubated for 72 hr before challenge with HIV^89.6R ^(MOI 0.7 on NP2-CD4-CXCR4). 48 hr post virus challenge, GFP expressing cells were recorded by the IN Cell Analyzer 1000 automated imaging system and analysed by IN Cell Developer software (GE Healthcare). Wells containing a GFP+ foci Z-score > 3 relative to the CB control (n = 16 per plate) were confirmed by visual inspection. 183/192 hits from the original screen were retested in triplicate. The top 52 hits were then further validated with four individual siRNAs per target. Targeted proteins rescuing infection by more than two out of four deconvoluted siRNAs were considered validated hits.

### siRNA transfection and infection with replication competent virus

HeLa-CD4 cells were seeded at 6.3 × 10^4 ^cells/well in 6-well plates. 72 hr after siRNA transfection (15 nM PAF1, CTR9, RTF1, LEO1/7.5 nM CDC73/30 nM all other siRNA), cells were challenged with HIV-1^89.6wt ^and HIV-1^NL4.3wt ^(MOI 0.2) for up to 5 hr. 1 hr prior to viral challenge, inhibitors (Raltegravir (1 μM), AZT (100 μM), SCH-D (1 μM) and AMD3100 (250 nM)) were added to control wells. Infection was assessed after 24-48 hr by intracellular p24 staining, real-time quantitative PCR (qPCR) or mRNA analysis.

### Western blot

SDS-PAGE separated cellular proteins, immobilised on Hybond-P PVDF membrane (GE Healthcare) were detected with the primary rabbit polyclonal antibody against (RbpAb-) PAF1, RbpAb-GAPDH and rat pAb-tubulin (Abcam) and a secondary horseradish peroxidise conjugated goat anti-rabbit/rat antibody (Promega). Proteins were visualised using a chemiluminescence kit (ECL, GE Healthcare).

### *In situ *immunostaining for p24 antigen

Infected cells were fixed with cold (-20°C) methanol:acetone (1:1), washed with PBS then immunostained for p24 using mouse anti-HIV-1 p24 monoclonal antibodies EVA365 and 366 (NIBSC) (1:50) or anti-HIV-2 patient human plasma (1:1000) (to detect HIV-2 and SIV infected cells), as previously described [68]. Infected cells were blue (regarded as foci of infection (FFU/ml)) and quantitated by light microscopy.

### First round *Alu-gag *PCR

DNA was extracted at various time points after infection with the QIAamp DNA Blood Mini Kit (QIAGEN). Integrated HIV-1 DNA was measured by nested PCR, as previously described [69].

### qPCR for HIV-1 DNA

The isolated DNA was subjected to qPCR to determine the number of early (negative strand strong stop) and late (*gag*) transcripts, normalised for cell number by genomic GAPDH as previously described.

### cDNA synthesis and mRNA analysis

Total HeLa-CD4 RNA was extracted an RNeasy Plant Mini Kit (QIAGEN) and cDNA was synthesised with Superscript III First Strand Synthesis System (Invitrogen), according to manufacturer's instructions. The cDNA produced was subjected to qPCR as described [69].

### Transfection of suspension cell lines

siRNA transfection of MM1 cells was performed using the AMAXA Nucleofector Kit V (Lonza Cologne AG) according to manufacturer's instructions. 48 hr following viral challenge, 20 ng/ml of phorbol 12-myristate 13-acetate (PMA) was added to induce cellular adherence prior to intracellular p24 staining.

### Cell cycle analysis

HeLa-CD4 cells were transfected with PAF1 or CB siRNA for 48 hr, or treated with nocodazole (40 ng/ml; Sigma) for 18 hr before cells were harvested. Cells were fixed with ice-cold 70% ethanol, washed and pelleted. Cells were RNaseA-treated (100 μg/ml; Sigma), stained with propidium iodide (50 μg/ml; Sigma) and analysed by flow cytometry (LSRII, BD Biosciences).

## Competing interests

The authors declare that they have no competing interests.

## Authors' contributions

LL developed, executed and validated the siRNA screen with help and advice from NMMO, ÁMcK, MTD, A-MB, VB and CLB (Figures 1, A1, 2, A3, A4). KMC and NMMO performed the biological and virological assays with assistance from HD and CP (Figures 3c, 4c-e, 5, 6) and LL (Figure 3a-b, 4a-b). ÁMcK conceived and guided the project with input from DHB and MTD. All authors contributed to writing the paper.

## Supplementary Material

Additional file 1 **Efficiency of siRNA knockdown on HIV infection**. siRNA knockdown of the HIV receptor CD4 and nuclear importin TNPO3 inhibits infection of HIV-1^8.2N ^by 95.8% and 93% respectively compared with CB control siRNA. Green, virus; red, cells. Click here for file

Additonal file 2 **Results of primary screen with Z-scores**. Positive results from primary screen are listed. Z-scores are given for all hits greater than 3SD from CB control siRNA. Click here for file

Additional file 3 **siRNA knockdown of 114 genes rescues HIV infection**. Infection of HIV^89.6R ^pseudotyped virus was rescued in HeLa-CD4 cells following siRNA knockdown of 114 genes. The y-axis denotes both the number of infectious viruses rescued (focus forming units/ml, FFU/ml; left) and fold rescue compared with the CB control (right). FFU/ml results are mean ± SD. Click here for file

Additional file 4 **Pathway analysis of most potent screen hits**. Ingenuity Pathway Analysis (IPA http://www.ingenuity.com) was performed on the validated screen hits showing the functions, if known, associated with the most potent. Click here for file

Additional file 5 **Network, functional and pathway analysis**. A list of 114 confirmed factors including heat map and GO terms. Click here for file
