# Supplementary figures and images for: A whole genome screen for HIV restriction factors
Source: Retrovirology. 2011 Nov 14;8:94. doi: 10.1186/1742-4690-8-94 (PMC3228845; doi:10.1186/1742-4690-8-94)

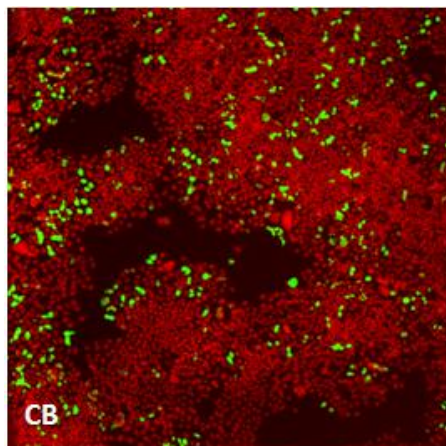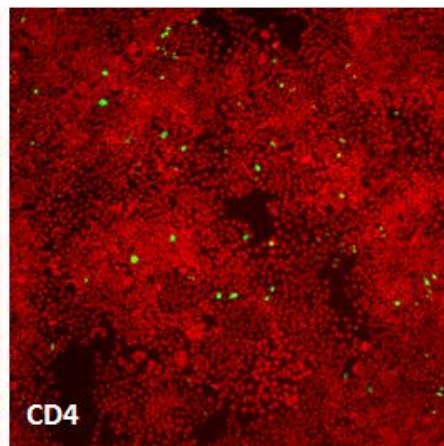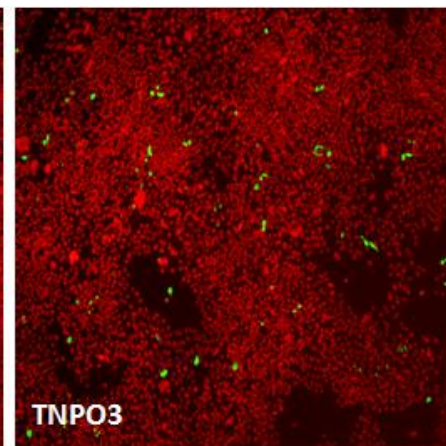

Supplement: Additional file 1 — Efficiency of siRNA knockdown on HIV infection. siRNA knockdown of the HIV receptor CD4 and nuclear importin TNPO3 inhibits infection of HIV-18.2N by 95.8% and 93% respectively compared with CB control siRNA. Green, virus; red, cells. [file 1742-4690-8-94-S1.PDF]

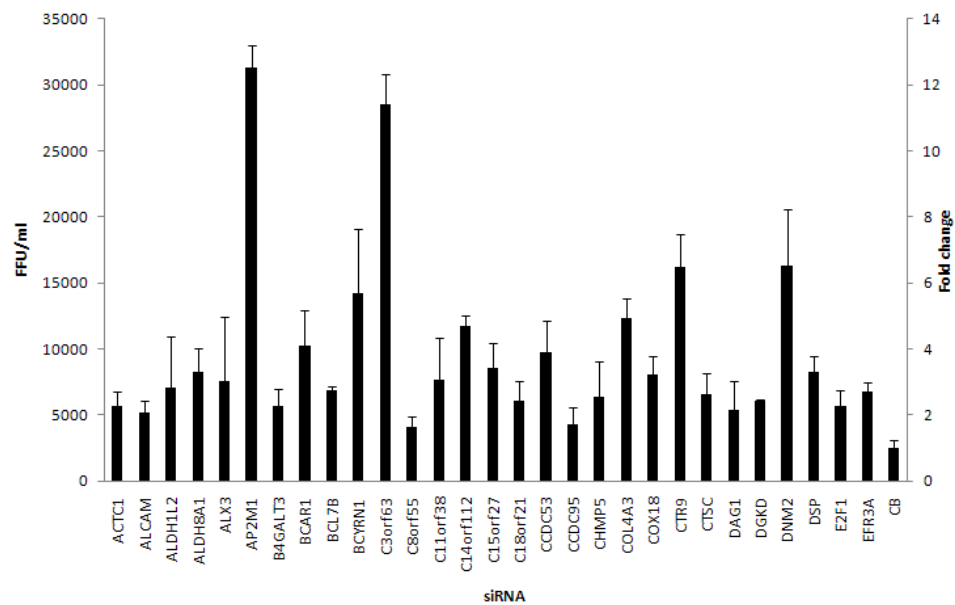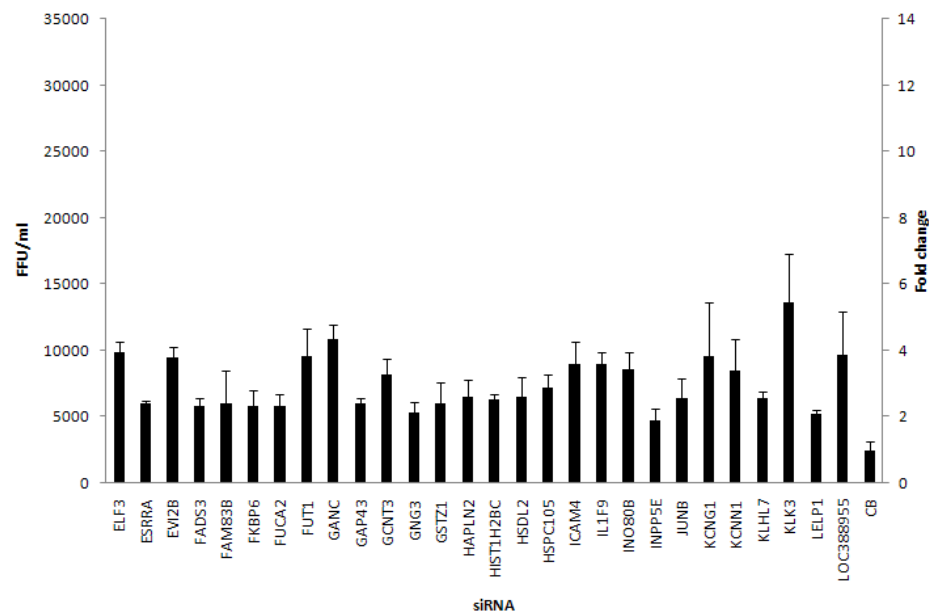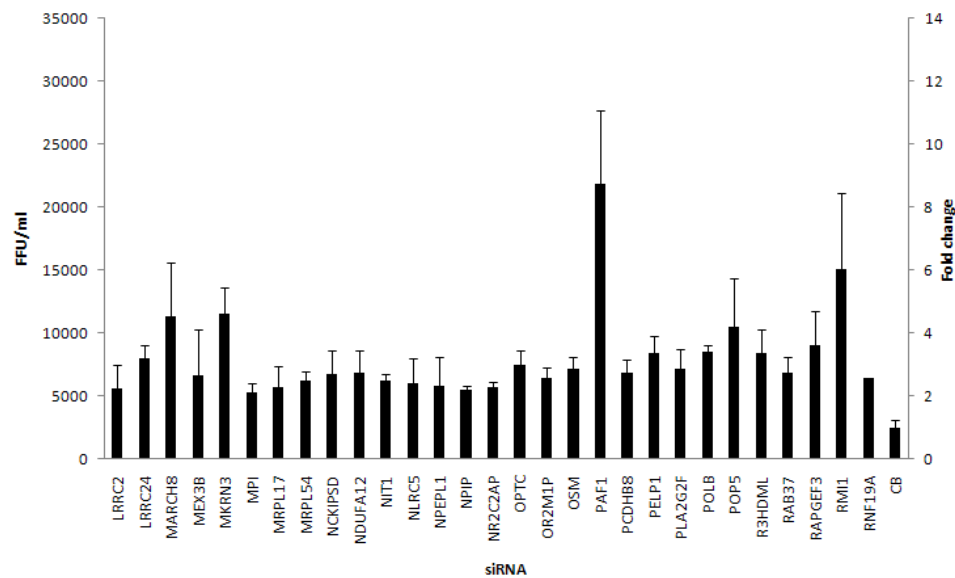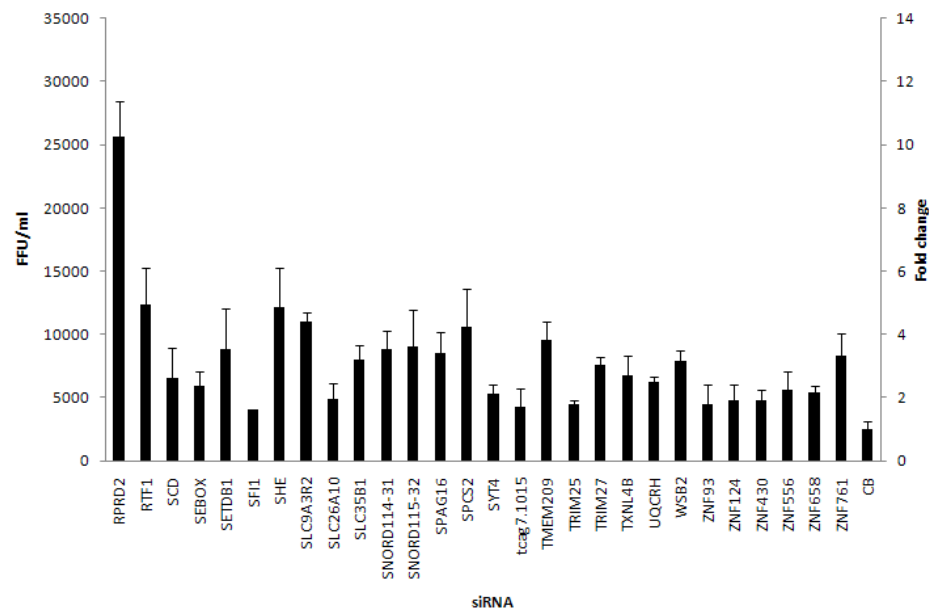

Supplement: Additional file 3 — siRNA knockdown of 114 genes rescues HIV infection. Infection of HIV89.6R pseudotyped virus was rescued in HeLa-CD4 cells following siRNA knockdown of 114 genes. The y-axis denotes both the number of infectious viruses rescued (focus forming units/ml, FFU/ml; left) and fold rescue compared with the CB control (right). FFU/ml results are mean ± SD. [file 1742-4690-8-94-S3.PDF]

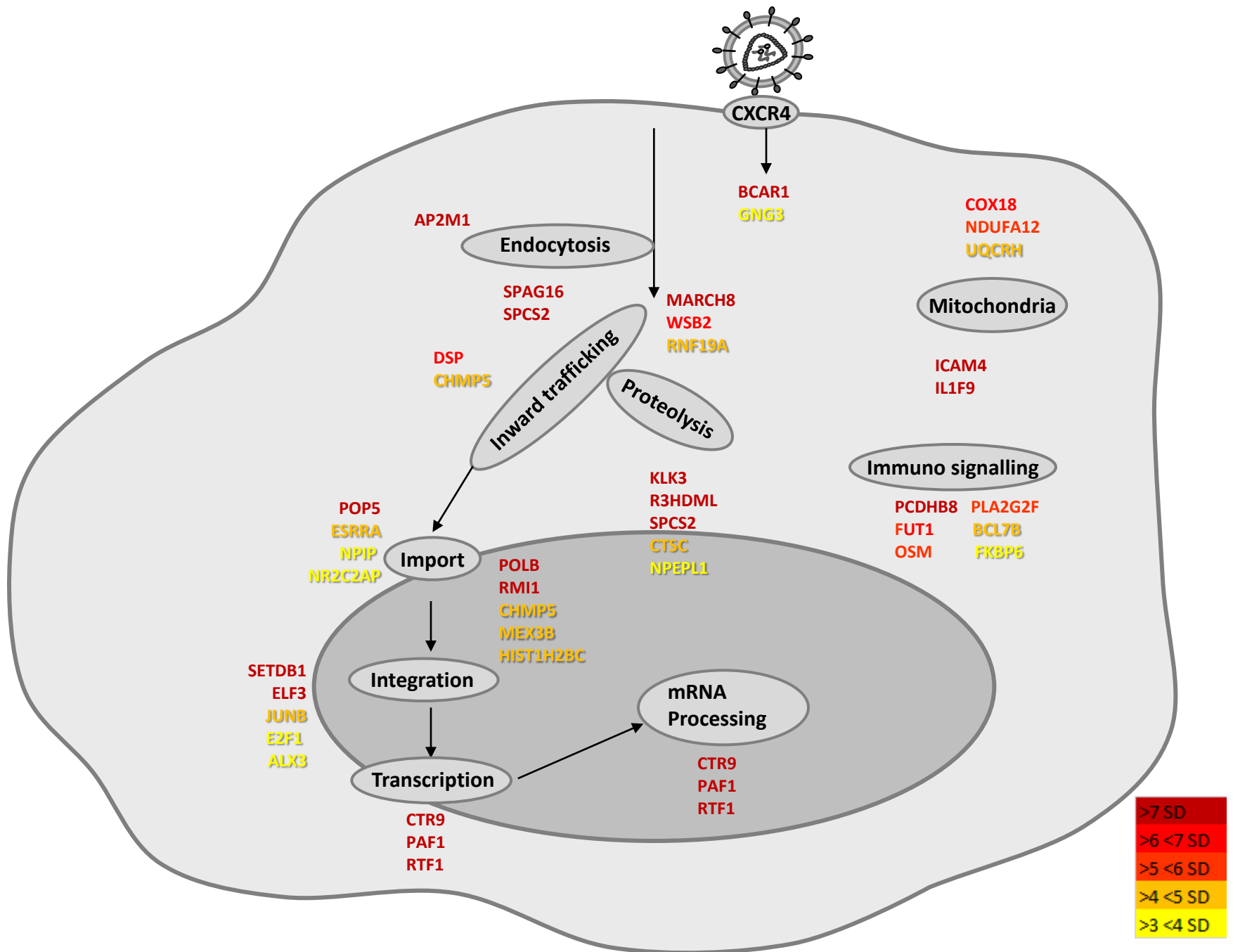

Supplement: Additional file 4 — Pathway analysis of most potent screen hits. Ingenuity Pathway Analysis (IPA http://www.ingenuity.com) was performed on the validated screen hits showing the functions, if known, associated with the most potent. [file 1742-4690-8-94-S4.PDF]
