# Supplementary material for: A whole genome screen for HIV restriction factors
Source: Retrovirology. 2011 Nov 14;8:94. doi: 10.1186/1742-4690-8-94 (PMC3228845; doi:10.1186/1742-4690-8-94)
Supplement: Additonal file 2 — Results of primary screen with Z-scores. Positive results from primary screen are listed. Z-scores are given for all hits greater than 3SD from CB control siRNA. [file 1742-4690-8-94-S2.PDF]

Gene ID	Gene Symbol	Gene Description	Z-score
1173	AP2M1	adaptor-related protein complex 2, mu 1 subunit	30.52491
220972	MARCH8	membrane-associated ring finger (C3HC4) 8	14.29118
3481	IGF2	insulin-like growth factor 2 (somatomedin A)	13.71141
7110	TMF1	TATA element modulatory factor 1	12.55185
57828	C19orf15	chromosome 19 open reading frame 15	11.3923
51241	C14orf112	chromosome 14 open reading frame 112	11.3923
3755	KCNG1	potassium voltage-gated channel, subfamily G, member 1	10.81253
56105	PCDHGA11	protocadherin gamma subfamily A, 11	10.81253
64577	ALDH8A1	aldehyde dehydrogenase 8 family, member A1	10.23275
27043	PELP1	proline, glutamate and leucine rich protein 1	10.23275
7626	ZNF75	zinc finger protein 75 (D8C6)	10.23275
219981	OR5A2	olfactory receptor, family 5, subfamily A, member 2	10.23275
4731	NDUFV3	NADH dehydrogenase (ubiquinone) flavoprotein 3, 10kDa	10.23275
91975	ZNF300	zinc finger protein 300	9.652973
80763	C12orf39	chromosome 12 open reading frame 39	9.652973
390061	OR51Q1	olfactory receptor, family 51, subfamily Q, member 1	9.652973
7681	MKRN3	makorin, ring finger protein, 3	9.073197
9351	SLC9A3R2	solute carrier family 9 (sodium/hydrogen exchanger), member 3 regulator 2	9.073197
56623	INPP5E	inositol polyphosphate-5-phosphatase, 72 kDa	9.073197
84914	ZNF587	zinc finger protein 587	9.073197
27163	NAAA	N-acylethanolamine acid amidase	9.073197
3188	HNRNPH2	heterogeneous nuclear ribonucleoprotein H2 (H')	9.073197
29123	ANKRD11	ankyrin repeat domain 11	8.493421
56882	CDC42SE1	CDC42 small effector 1	8.493421
284058	KIAA1267	KIAA1267	8.493421
79998	ANKRD53	ankyrin repeat domain 53	8.493421
339168	TMEM95	transmembrane protein 95	8.493421
390162	OR5M9	olfactory receptor, family 5, subfamily M, member 9	8.493421
3012	HIST1H2AE	histone cluster 1, H2ae	8.493421
354	KLK3	kallikrein-related peptidase 3	7.913645
9564	BCAR1	breast cancer anti-estrogen resistance 1	7.913645

160428	ALDH1L2	aldehyde dehydrogenase 1 family, member L2	7.913645
1007	CDH9	cadherin 9, type 2 (T1-cadherin)	7.913645
692210	SNORD93	small nucleolar RNA, C/D box 93	7.913645
5413	SEPT5	septin 5	7.913645
2519	FUCA2	fucosidase, alpha-L- 2, plasma	7.333869
2101	ESRRA	estrogen-related receptor alpha	7.333869
57589	KIAA1432	KIAA1432	7.333869
91582	RPS19BP1	ribosomal protein S19 binding protein 1	7.333869
9848	MFAP3L	microfibrillar-associated protein 3-like	7.333869
252946	CYorf16	chromosome Y open reading frame 16	7.333869
282775	OR5J2	olfactory receptor, family 5, subfamily J, member 2	7.333869
28952	CCDC22	coiled-coil domain containing 22	7.333869
127700	C1orf102	chromosome 1 open reading frame 102	7.333869
9080	CLDN9	claudin 9	7.333869
84263	HSDL2	hydroxysteroid dehydrogenase like 2	6.754093
9869	SETDB1	SET domain, bifurcated 1	6.754093
326624	RAB37	RAB37, member RAS oncogene family	6.754093
23158	TBC1D9	TBC1 domain family, member 9 (with GRAM domain)	6.754093
23582	CCNDBP1	cyclin D-type binding-protein 1	6.754093
58484	NLRC4	NLR family, CARD domain containing 4	6.754093
81606	LBH	limb bud and heart development homolog (mouse)	6.754093
8356	HIST1H3J	histone cluster 1, H3j	6.754093
123591	C15orf27	chromosome 15 open reading frame 27	6.174317
940	CD28	CD28 molecule	6.174317
79781	IQCA	IQ motif containing with AAA domain	6.174317
692111	SNORD71	small nucleolar RNA, C/D box 71	6.174317
246329	STAC3	SH3 and cysteine rich domain 3	6.174317
4821	NKX2-2	NK2 homeobox 2	6.174317
9487	PIGL	phosphatidylinositol glycan anchor biosynthesis, class L	6.174317
56953	NT5M	5',3'-nucleotidase, mitochondrial	6.174317
7089	TLE2	transducin-like enhancer of split 2 (E(sp1) homolog, Drosophila)	6.174317
26528	DAZAP1	DAZ associated protein 1	6.174317
79716	NPEPL1	aminopeptidase-like 1	5.594541

5008	OSM	oncostatin M	5.594541
5423	POLB	polymerase (DNA directed), beta	5.594541
4018	LPA	lipoprotein, Lp(a)	5.594541
84206	MEX3B	mex-3 homolog B (C. elegans)	5.594541
3909	LAMA3	laminin, alpha 3	5.594541
1605	DAG1	dystroglycan 1 (dystrophin-associated glycoprotein 1)	5.594541
4161	MC5R	melanocortin 5 receptor	5.594541
148229	ATP8B3	ATPase, class I, type 8B, member 3	5.594541
80131	LRRC8E	leucine rich repeat containing 8 family, member E	5.594541
170712	COX7B2	cytochrome c oxidase subunit VIIb2	5.594541
403282	OR6C65	olfactory receptor, family 6, subfamily C, member 65	5.594541
133558	FLJ40243	hypothetical protein FLJ40243	5.594541
128497	C20orf165	chromosome 20 open reading frame 165	5.594541
100033815	SNORD115-41	small nucleolar RNA, C/D box 115-41	5.594541
2553	GABPB2	GA binding protein transcription factor, beta subunit 2	5.594541
64711	HS3ST6	heparan sulfate (glucosamine) 3-O-sulfotransferase 6	5.594541
389827	RP13-388O5.1	transmembrane protein 8-like	5.594541
6358	CCL14	chemokine (C-C motif) ligand 14	5.594541
2785	GNG3	guanine nucleotide binding protein (G protein), gamma 3	5.014765
25897	RNF19A	ring finger protein 19A	5.014765
4193	MDM2	Mdm2 p53 binding protein homolog (mouse)	5.014765
1285	COL4A3	collagen, type IV, alpha 3 (Goodpasture antigen)	5.014765
5987	TRIM27	tripartite motif-containing 27	5.014765
7225	TRPC6	transient receptor potential cation channel, subfamily C, member 6	5.014765
3726	JUNB	jun B proto-oncogene	5.014765
2595	GANC	glucosidase, alpha; neutral C	5.014765
64600	PLA2G2F	phospholipase A2, group IIF	5.014765
23162	MAPK8IP3	mitogen-activated protein kinase 8 interacting protein 3	5.014765
55001	TTC22	tetratricopeptide repeat domain 22	5.014765
10174	SORBS3	sorbin and SH3 domain containing 3	5.014765
54752	FNDC8	fibronectin type III domain containing 8	5.014765
9121	SLC16A5	solute carrier family 16, member 5 (monocarboxylic acid transporter 6)	5.014765
6687	SPG7	spastic paraplegia 7 (pure and complicated autosomal recessive)	5.014765

3217	HOXB7	homeobox B7	5.014765
60680	BRUNOL5	bruno-like 5, RNA binding protein (Drosophila)	5.014765
28999	KLF15	Kruppel-like factor 15	5.014765
84626	KRBA1	KRAB-A domain containing 1	5.014765
392979	tcag7.23	similar to ribosomal protein L18; 60S ribosomal protein L18	5.014765
2954	GSTZ1	glutathione transferase zeta 1 (maleylacetoacetate isomerase)	4.434988
56300	IL1F9	interleukin 1 family, member 9	4.434988
2523	FUT1	fucosyltransferase 1 (galactoside 2-alpha-L-fucosyltransferase, H blood group)	4.434988
1869	E2F1	E2F transcription factor 1	4.434988
3780	KCNN1	potassium intermediate/small conductance calcium-activated channel, subfamily N, member 1	4.434988
48	ACO1	aconitase 1, soluble	4.434988
57679	ALS2	amyotrophic lateral sclerosis 2 (juvenile)	4.434988
50808	AK3	adenylate kinase 3	4.434988
3646	EIF3E	eukaryotic translation initiation factor 3, subunit E	4.434988
79665	DHX40	DEAH (Asp-Glu-Ala-His) box polypeptide 40	4.434988
9938	ARHGAP25	Rho GTPase activating protein 25	4.434988
8537	BCAS1	breast carcinoma amplified sequence 1	4.434988
55270	NUDT15	nudix (nucleoside diphosphate linked moiety X)-type motif 15	4.434988
2498	FTHL3	ferritin, heavy polypeptide-like 3	4.434988
83899	KRTAP9-2	keratin associated protein 9-2	4.434988
93129	ORAI3	ORAI calcium release-activated calcium modulator 3	4.434988
5697	PYY	peptide YY	4.434988
339240	LOC339240	keratin pseudogene	4.434988
84317	CCDC115	coiled-coil domain containing 115	4.434988
390110	LOC390110	hypothetical protein	4.434988
390037	OR52I1	olfactory receptor, family 52, subfamily I, member 1	4.434988
196383	RILPL2	Rab interacting lysosomal protein-like 2	4.434988
8817	FGF18	fibroblast growth factor 18	4.434988
10410	IFITM3	interferon induced transmembrane protein 3 (1-8U)	4.434988
26636	OR7E37P	olfactory receptor, family 7, subfamily E, member 37 pseudogene	4.434988
8175	SF3A2	splicing factor 3a, subunit 2, 66kDa	4.434988
7388	UQCRH	ubiquinol-cytochrome c reductase hinge protein	3.855212
5054	SERPINE1	serpin peptidase inhibitor, clade E (nexin, plasminogen activator inhibitor type 1), member 1	3.855212

79442	LRRC2	leucine rich repeat containing 2	3.855212
5002	SLC22A18	solute carrier family 22, member 18	3.855212
1075	CTSC	cathepsin C	3.855212
54957	TXNL4B	thioredoxin-like 4B	3.855212
2778	GNAS	GNAS complex locus	3.855212
1832	DSP	desmoplakin	3.855212
126382	NR2C2AP	nuclear receptor 2C2-associated protein	3.855212
50846	DHH	desert hedgehog homolog (Drosophila)	3.855212
55629	PNRC2	proline-rich nuclear receptor coactivator 2	3.855212
55971	BAIAP2L1	BAI1-associated protein 2-like 1	3.855212
79623	GALNT14	UDP-N-acetyl-alpha-D-galactosamine:polypeptide N-acetylgalactosaminyltransferase 14 (GalNAc-T14)	3.855212
160728	SLC5A8	solute carrier family 5 (iodide transporter), member 8	3.855212
57062	DDX24	DEAD (Asp-Glu-Ala-Asp) box polypeptide 24	3.855212
145173	B3GALTL	beta 1,3-galactosyltransferase-like	3.855212
57699	CPNE5	copine V	3.855212
221016	CCDC7	coiled-coil domain containing 7	3.855212
57325	CSRP2BP	CSRP2 binding protein	3.855212
1615	DARS	aspartyl-tRNA synthetase	3.855212
2767	GNA11	guanine nucleotide binding protein (G protein), alpha 11 (Gq class)	3.855212
26301	GBGT1	globoside alpha-1,3-N-acetylgalactosaminyltransferase 1	3.855212
5827	PXMP2	peroxisomal membrane protein 2, 22kDa	3.855212
114299	PALM2	paralemmin 2	3.855212
83864	TTTY9A	testis-specific transcript, Y-linked 9A	3.855212
390429	OR4N2	olfactory receptor, family 4, subfamily N, member 2	3.855212
10578	GNLY	granulysin	3.855212
89790	SIGLEC10	sialic acid binding Ig-like lectin 10	3.275436
55884	WSB2	WD repeat and SOCS box-containing 2	3.275436
10874	NMU	neuromedin U	3.275436
11219	TREX2	three prime repair exonuclease 2	3.275436
7706	TRIM25	tripartite motif-containing 25	3.275436
4860	NP	nucleoside phosphorylase	3.275436
26272	FBXO4	F-box protein 4	3.275436
54332	GDAP1	ganglioside-induced differentiation-associated protein 1	3.275436

60529	ALX4	aristaless-like homeobox 4	3.275436
214	ALCAM	activated leukocyte cell adhesion molecule	3.275436
135250	RAET1E	retinoic acid early transcript 1E	3.275436
3673	ITGA2	integrin, alpha 2 (CD49B, alpha 2 subunit of VLA-2 receptor)	3.275436
3061	HCRTR1	hypocretin (orexin) receptor 1	3.275436
8527	DGKD	diacylglycerol kinase, delta 130kDa	3.275436
10036	CHAF1A	chromatin assembly factor 1, subunit A (p150)	3.275436
1291	COL6A1	collagen, type VI, alpha 1	3.275436
11123	RCAN3	RCAN family member 3	3.275436
256880	LOC256880	hypothetical LOC256880	3.275436
1665	DHX15	DEAH (Asp-Glu-Ala-His) box polypeptide 15	3.275436
199920	C1orf168	chromosome 1 open reading frame 168	3.275436
55340	GIMAP5	GTPase, IMAP family member 5	3.275436
81556	C15orf44	chromosome 15 open reading frame 44	3.275436
122786	FRMD6	FERM domain containing 6	3.275436
80324	PUS1	pseudouridylate synthase 1	3.275436
56146	PCDHA2	protocadherin alpha 2	3.275436
81856	ZNF611	zinc finger protein 611	3.275436
11182	SLC2A6	solute carrier family 2 (facilitated glucose transporter), member 6	3.275436
9442	MED27	mediator complex subunit 27	3.275436
81532	HCCA2	HCCA2 protein	3.275436
93210	PERLD1	per1-like domain containing 1	3.275436
55762	ZNF701	zinc finger protein 701	3.275436
130367	SGPP2	sphingosine-1-phosphate phosphotase 2	3.275436
80199	FUZ	fuzzy homolog (Drosophila)	3.275436
51270	TFDP3	transcription factor Dp family, member 3	3.275436
54093	SETD4	SET domain containing 4	3.275436
1611	DAP	death-associated protein	3.275436
23126	POGZ	pogo transposable element with ZNF domain	3.275436
