# Supplementary material for: A whole genome screen for HIV restriction factors
Source: Retrovirology. 2011 Nov 14;8:94. doi: 10.1186/1742-4690-8-94 (PMC3228845; doi:10.1186/1742-4690-8-94)
Supplement: Additional file 5 — Network, functional and pathway analysis. A list of 114 confirmed factors including heat map and GO terms. [file 1742-4690-8-94-S5.PDF]

	Gene ID	GeneSymbol	Gene Description	Triplicates	GO Molecular Function
1	1173	AP2M1	adaptor-related protein complex 2, mu 1 subunit		
2	23272	C3orf63	chromosome 3 open reading frame 63		
3	23248	RPRD2	regulation of nuclear pre-mRNA domain containing 2		protein binding;kinase inhibitor activity;kinase regulator activity
4	1785	DNM2	dynamin 2		GTPase activity;structural constituent of
5	80010	RMI1	RMI1, RecQ mediated genome instability 1, homolog (S.		
6	618	BCYRN1	brain cytoplasmic RNA 1, Bc1 analog (mouse)		
7	54623	PAF1	Paf1, RNA polymerase II associated factor, homolog (S.		
8	354	KLK3	kallikrein-related peptidase 3		peptidase activity
9	23168	RTF1	Rtf1, Paf1/RNA polymerase II complex component, homolog (S. cerevisiae)		
10	51241	C14orf112	COX16		
11	7681	MKRN3	makorin, ring finger protein, 3		ubiquitin-protein ligase activity
12	220972	MARCH8	membrane-associated ring finger (C3HC4) 8		ubiquitin-protein ligase activity
13	9351	SLC9A3R2	solute carrier family 9 (sodium/hydrogen exchanger), member 3 regulator 2		
14	51367	POP5	processing of precursor 5, ribonuclease P/MRP subunit (S.		
15	9646	CTR9	Ctr9, Paf1/RNA polymerase II complex component, homolog (S. cerevisiae)		
16	9564	BCAR1	breast cancer anti-estrogen resistance 1		structural constituent of cytoskeleton
17	3755	KCNG1	potassium voltage-gated channel, subfamily G, member 1		cation transmembrane transporter activity;voltage-gated potassium channel activity;cation channel activity
18	100033806	SNORD115-32	small nucleolar RNA, C/D box 115-32		
19	3386	ICAM4	intercellular adhesion molecule 4 (Landsteiner-Wiener		receptor activity;receptor binding
20	83444	ZNHIT4	zinc finger, HIT type 4		
21	79582	SPAG16	sperm associated antigen 16		structural constituent of cytoskeleton
22	27043	PELP1	proline, glutamate and leucine rich protein 1		receptor activity;extracellular matrix structural constituent;receptor binding
23	140902	R3HDML	R3H domain containing-like		
24	388561	ZNF761	zinc finger protein 761		DNA binding;transcription factor activity
25	64577	ALDH8A1	aldehyde dehydrogenase 8 family, member A1		oxidoreductase activity
26	1285	COL4A3	collagen, type IV, alpha 3 (Goodpasture antigen)		receptor activity;extracellular matrix structural constituent;receptor binding
27	126669	SHE	Src homology 2 domain containing E		
28	5987	TRIM27	tripartite motif-containing 27		ubiquitin-protein ligase activity;structural constituent of cytoskeleton;RNA binding;cytoskeletal protein binding
29	26254	OPTC	opticin		receptor activity
30	93517	HSPC105	NAD(P) dependent steroid dehydrogenase-like		oxidoreductase activity;hydro-lyase activity;racemase and epimerase activity
31	160428	ALDH1L2	aldehyde dehydrogenase 1 family, member L2		oxidoreductase activity

>7 SD
>6 <7 SD
>5 <6 SD
>4 <5 SD
>3 <4 SD
>2 <3 SD
<2 SD

32	9789	SPCS2	signal peptidase complex subunit 2 homolog (S. cerevisiae)		protein binding
33	56128	PCDHB8	protocadherin beta 8		G-protein coupled receptor activity;calcium ion binding
34	51517	NCKIPSD	NCK interacting protein with SH3 domain		
35	2595	GANC	glucosidase, alpha; neutral C		hydrolase activity, hydrolyzing O-glycosyl compounds
36	388955	LOC388955	PX19 protein pseudogene (PRELID1)		
37	2124	EVI2B	ecotropic viral integration site 2B		
38	1999	ELF3	E74-like factor 3 (ets domain transcription factor, epithelial-specific)		DNA binding;receptor binding;transcription factor activity
39	56300	IL1F9	interleukin 1 family, member 9		receptor binding
40	767612	SNORD114-31	small nucleolar RNA, C/D box 114-31		
41	9869	SETDB1	SET domain, bifurcated 1		methyltransferase activity;DNA binding
42	84928	TMEM209	transmembrane protein 209		
43	5423	POLB	polymerase (DNA directed), beta		DNA-directed DNA polymerase activity;nucleic acid binding
44	51019	CCDC53	coiled-coil domain containing 53		
45	123591	C15orf27	chromosome 15 open reading frame 27		
46	70	ACTC1	actin, alpha, cardiac muscle 1		structural constituent of cytoskeleton
47	1832	DSP	desmoplakin		structural constituent of cytoskeleton;cytoskeletal protein
48	9245	GCNT3	glucosaminyl (N-acetyl) transferase 3, mucin type		transferase activity, transferring glycosyl groups
49	80032	ZNF556	zinc finger protein 556		DNA binding;transcription factor activity
50	285521	COX18	COX18 cytochrome c oxidase assembly homolog (S.		transmembrane transporter activity
51	2523	FUT1	fucosyltransferase 1 (galactoside 2-alpha-L-fucosyltransferase, H blood group)		transferase activity, transferring glycosyl groups
52	3780	KCNN1	potassium intermediate/small conductance calcium-activated channel, subfamily N, member 1		cation transmembrane transporter activity;voltage-gated potassium channel activity;cation channel activity
53	10237	SLC35B1	solute carrier family 35, member B1		transmembrane transporter activity
54	10411	RAPGEF3	Rap guanine nucleotide exchange factor (GEF) 3		protein binding;small GTPase regulator activity;guanyl-nucleotide exchange factor activity
55	55884	WSB2	WD repeat and SOCS box-containing 2		
56	26149	ZNF658	zinc finger protein 658		DNA binding;transcription factor activity
57	441381	LRRC24	leucine rich repeat containing 24		receptor activity
58	64600	PLA2G2F	phospholipase A2, group IIF		hydrolase activity, acting on ester bonds
59	5008	OSM	oncostatin M		receptor binding
60	65012	SLC26A10	solute carrier family 26, member 10		
61	55967	NDUFA12	NADH dehydrogenase (ubiquinone) 1 alpha subcomplex,		oxidoreductase activity
62	326624	RAB37	RAB37, member RAS oncogene family		GTPase activity;protein binding
63	23167	EFR3A	EFR3 homolog A (S. cerevisiae)		
64	7678	ZNF124	zinc finger protein 124		DNA binding;transcription factor activity
65	80264	ZNF430	zinc finger protein 430		DNA binding;transcription factor activity
66	1075	CTSC	cathepsin C		peptidase activity
67	56623	INPP5E	inositol polyphosphate-5-phosphatase, 72 kDa		
68	399967	C11orf38	prostate and testis expressed 2 (PATE2)		
69	60484	HAPLN2	hyaluronan and proteoglycan link protein 2		

70	84263	HSDL2	hydroxysteroid dehydrogenase like 2		oxidoreductase activity
71	9275	BCL7B	B-cell CLL/lymphoma 7B		
72	388762	OR2M1P	olfactory receptor, family 2, subfamily M, member 1		
73	25897	RNF19A	ring finger protein 19A		ubiquitin-protein ligase activity;DNA binding;transcription factor activity;transcription cofactor activity
74	54957	TXNL4B	thioredoxin-like 4B		
75	84206	MEX3B	mex-3 homolog B (C. elegans)		ubiquitin-protein ligase activity;RNA binding
76	7706	TRIM25	tripartite motif-containing 25		ubiquitin-protein ligase activity;structural constituent of cytoskeleton;nucleic acid binding;cytoskeletal protein
77	4817	NIT1	nitrilase 1		hydrolase activity
78	3726	JUNB	jun B proto-oncogene		DNA binding;transcription factor activity
79	7388	UQCRH	ubiquinol-cytochrome c reductase hinge protein		oxidoreductase activity
80	81931	ZNF93	zinc finger protein 93		DNA binding;transcription factor activity
81	116541	MRPL54	mitochondrial ribosomal protein L54		
82	83608	C18orf21	chromosome 18 open reading frame 21		
83	8527	DGKD	diacylglycerol kinase, delta 130kDa		kinase activity
84	222584	FAM83B	family with sequence similarity 83, member B		
85	84166	NLRC5	NLR family, CARD domain containing 5		
86	51510	CHMP5	chromatin modifying protein 5		
87	2101	ESRRA	estrogen-related receptor alpha		ligand-dependent nuclear receptor activity;DNA binding;transcription factor activity
88	2596	GAP43	growth associated protein 43		
89	2954	GSTZ1	glutathione transferase zeta 1 (maleylacetoacetate isomerase)		oxidoreductase activity;transferase activity;racemase and epimerase activity;structural constituent of cytoskeleton;anion channel activity;translation factor activity, nucleic acid binding;receptor binding;translation
90	283899	CCDC95	coiled-coil domain containing 95		
91	286016	TPI1P2	triosephosphate isomerase 1 pseudogene 2		
92	8347	HIST1H2BC	histone cluster 1, H2bc		DNA binding
93	645832	SEBOX	SEBOX homeobox		DNA binding;transcription factor activity
94	8468	FKBP6	FK506 binding protein 6, 36kDa		isomerase activity
95	2519	FUCA2	fucosidase, alpha-L- 2, plasma		hydrolase activity, hydrolyzing N-glycosyl compounds
96	55975	KLHL7	kelch-like 7 (Drosophila)		peptidase activity;structural constituent of cytoskeleton;DNA binding;cytoskeletal protein binding;transcription factor activity;transcription cofactor
97	257	ALX3	aristaless-like homeobox 3		DNA binding;transcription factor activity
98	8703	B4GALT3	UDP-Gal:betaGlcNAc beta 1,4- galactosyltransferase,		transferase activity, transferring glycosyl groups
99	3995	FADS3	fatty acid desaturase 3		
100	126382	NR2C2AP	nuclear receptor 2C2-associated protein		
101	51337	C8orf55	chromosome 8 open reading frame 55		
102	79442	LRRC2	leucine rich repeat containing 2		adenylate cyclase activity;receptor binding;kinase regulator
103	9284	NPIP	nuclear pore complex interacting protein		

104	9814	SFI1	Sfi1 homolog, spindle assembly associated (yeast)		
105	63875	MRPL17	mitochondrial ribosomal protein L17		structural constituent of ribosome;nucleic acid binding
106	1605	DAG1	dystroglycan 1 (dystrophin-associated glycoprotein 1)		receptor activity
107	2785	GNG3	guanine nucleotide binding protein (G protein), gamma 3		GTPase activity;protein binding
108	4351	MPI	mannose phosphate isomerase		isomerase activity
109	79716	NPEPL1	aminopeptidase-like 1		peptidase activity
110	6860	SYT4	synaptotagmin IV		
111	149018	LELP1	late cornified envelope-like proline-rich 1		structural molecule activity
112	6319	SCD	stearoyl-CoA desaturase (delta-9-desaturase)		oxidoreductase activity
113	214	ALCAM	activated leukocyte cell adhesion molecule		receptor activity
114	1869	E2F1	E2F transcription factor 1		DNA binding;transcription factor activity
115	3673	ITGA2	integrin, alpha 2 (CD49B, alpha 2 subunit of VLA-2		
116	387264	KRTAP5-1	keratin associated protein 5-1		structural constituent of cytoskeleton
117	253982	ASPHD1	aspartate beta-hydroxylase domain containing 1		oxidoreductase activity
118	51475	CABP2	calcium binding protein 2		calcium ion binding;calmodulin binding
119	2778	GNAS	GNAS complex locus		GTPase activity;protein binding
120	5002	SLC22A18	solute carrier family 22, member 18		transmembrane transporter activity
121	48	ACO1	aconitase 1, soluble		hydro-lyase activity
122	25832	NBPF14	neuroblastoma breakpoint family, member 14		
123	9487	PIGL	phosphatidylinositol glycan anchor biosynthesis, class L		deacetylase activity
124	164127	C1orf65	chromosome 1 open reading frame 65		
125	3241	HPCAL1	hippocalcin-like 1		calcium ion binding;calmodulin binding;small GTPase regulator activity
126	29124	LGALS13	lectin, galactoside-binding, soluble, 13 (galectin 13)		receptor binding
127	4193	MDM2	Mdm2 p53 binding protein homolog (mouse)		ubiquitin-protein ligase activity
128	79570	NKAIN1	Na+/K+ transporting ATPase interacting 1		
129	26272	FBXO4	F-box protein 4		
130	5730	PTGDS	prostaglandin D2 synthase 21kDa (brain)		isomerase activity
131	50846	DHH	desert hedgehog homolog (Drosophila)		peptidase activity;receptor binding
132	54332	GDAP1	ganglioside-induced differentiation-associated protein 1		oxidoreductase activity;transferase activity;racemase and epimerase activity;structural constituent of cytoskeleton;anion channel activity;translation factor activity, nucleic acid binding;receptor binding;translation
133	60529	ALX4	aristaless-like homeobox 4		DNA binding;transcription factor activity
134	3909	LAMA3	laminin, alpha 3		
135	201254	STRA13	stimulated by retinoic acid 13 homolog (mouse)		
136	135250	RAET1E	retinoic acid early transcript 1E		receptor activity
137	115509	ZNF689	zinc finger protein 689		DNA binding;transcription factor activity
138	84232	MAF1	MAF1 homolog (S. cerevisiae)		DNA binding;transcription factor activity
139	4161	MC5R	melanocortin 5 receptor		G-protein coupled receptor activity
140	27433	TOR2A	torsin family 2, member A		

141	103910	MRLC2	myosin regulatory light chain MRLC2		structural constituent of cytoskeleton;calcium ion binding;calmodulin binding
142	55256	ADI1	acireductone dioxygenase 1		oxidoreductase activity
143	7225	TRPC6	transient receptor potential cation channel, subfamily C,		ion channel activity
144	3061	HCRTR1	hypocretin (orexin) receptor 1		G-protein coupled receptor activity
145	26578	OSTF1	osteoclast stimulating factor 1		
146	55629	PNRC2	proline-rich nuclear receptor coactivator 2		
147	55362	TMEM63B	transmembrane protein 63B		
148	164668	APOBEC3H	apolipoprotein B mRNA editing enzyme, catalytic polypeptide-like 3H		hydrolase activity;deaminase activity;nucleic acid binding
149	55135	WDR79	WD repeat domain 79		
150	55971	BAIAP2L1	BAI1-associated protein 2-like 1		receptor activity
151	196483	FAM86A	family with sequence similarity 86, member A		
152	2495	FTH1	ferritin, heavy polypeptide 1		
153	286053	NSMCE2	non-SMC element 2, MMS21 homolog (S. cerevisiae)		
154	641372	ACOT6	acyl-CoA thioesterase 6		
155	50808	AK3	adenylate kinase 3		kinase activity
156	57679	ALS2	amyotrophic lateral sclerosis 2 (juvenile)		ligase activity;nucleic acid binding;chromatin binding;protein binding;small GTPase regulator activity;guanyl-nucleotide exchange factor activity
157	940	CD28	CD28 molecule		receptor activity;receptor binding
158	729359	KIAA1881	KIAA1881		
159	148418	SAMD13	sterile alpha motif domain containing 13		DNA binding;chromatin binding;transcription factor activity
160	91828	C14orf73	chromosome 14 open reading frame 73		
161	29057	FAM156A	family with sequence similarity 156, member A		
162	55146	ZDHHC4	zinc finger, DHHC-type containing 4		
163	80818	ZNF436	zinc finger protein 436		DNA binding;transcription factor activity
164	114795	TMEM132B	transmembrane protein 132B		
165	81853	TMEM14B	transmembrane protein 14B		
166	1211	CLTA	clathrin, light chain (Lca)		
167	3898	LAD1	ladinin 1		structural molecule activity
168	23162	MAPK8IP3	mitogen-activated protein kinase 8 interacting protein 3		protein binding;kinase regulator activity
169	162515	SLC16A11	solute carrier family 16, member 11 (monocarboxylic acid transporter 11)		transmembrane transporter activity
170	2739	GLO1	glyoxalase I		lyase activity
171	56104	PCDHGB1	protocadherin gamma subfamily B, 1		
172	57829	ZP4	zona pellucida glycoprotein 4		receptor activity
173	55049	C19orf60	chromosome 19 open reading frame 60		
174	155400	NSUN5B	NOL1/NOP2/Sun domain family, member 5B		
175	5924	RASGRF2	Ras protein-specific guanine nucleotide-releasing factor 2		protein binding;small GTPase regulator activity;guanyl-nucleotide exchange factor activity
176	338069	ST7OT4	ST7 overlapping transcript 4 (non-coding RNA)		

177	26520	TIMM9	translocase of inner mitochondrial membrane 9 homolog		
178	10874	NMU	neuromedin U		
179	5054	SERPINE1	serpin peptidase inhibitor, clade E (nexin, plasminogen activator inhibitor type 1), member 1		protein binding;peptidase inhibitor activity
180	4018	LPA	lipoprotein, Lp(a)		peptidase activity;calcium ion binding;receptor binding;calmodulin binding;calcium-dependent phospholipid binding;peptidase inhibitor activity
181	862	RUNX1T1	runt-related transcription factor 1; translocated to, 1 (cyclin D-related)		DNA binding;transcription factor activity;transcription cofactor activity
182	337970	KRTAP19-3	keratin associated protein 19-3		DNA binding;transcription factor activity
183	81442	OR6N2	olfactory receptor, family 6, subfamily N, member 2		
